# Inconsistent Capacity Assessment Access for Adult Protective Services: National Survey Results

**DOI:** 10.1093/geroni/igaa057.152

**Published:** 2020-12-16

**Authors:** Theresa Sivers-Teixeira, Kelly Sadamitsu, Gregory Stevens, Christina Penate, Bonnie Olsen

**Affiliations:** Keck School of Medicine of USC, Alhambra, California, United States

## Abstract

Throughout the country, access to professional capacity assessments is inconsistent. Capacity assessments are often a critical factor in case management of elder abuse investigations. This unmet need for many Adult Protective Services (APS) programs hampers their ability to protect older adults from mistreatment and preserve self-determination when appropriate. In June 2019, electronic surveys regarding access to capacity assessments were sent to APS leadership in all 58 California counties. A similar survey is planned for each US state in June 2020. In California, with 100% response rate, 53% of counties had no access to assessments and 56.1% had no funding for assessments. Most assessments were completed by physicians or psychologists. Seventy-three percent of counties reported that primary care physicians complete requests for capacity declarations less than half the time. Physicians decline to complete capacity declarations because they don’t know how to do the assessments (22.4%) and are concerned about being called into court (28.5%). Findings from the national survey will be presented along with maps illustrating capacity assessment accessibility. Factors that appear to influence accessibility positively (forensic centers) and negatively (lack of funding and lack of trained evaluators) will be discussed along with policy implications.

